# Epidemiological surveillance of capybaras and ticks on warning area for Brazilian spotted fever

**DOI:** 10.14202/vetworld.2015.1143-1149

**Published:** 2015-09-30

**Authors:** José Brites-Neto, Jardel Brasil, Keila Maria Roncato Duarte

**Affiliations:** 1 Department of Epidemiological Surveillance, Secretariat of Health, Americana, São Paulo, Brazil; 2 Department of Genetics and Animal Reproduction, Institute of Animal Science, Nova Odessa, São Paulo, Brazil

**Keywords:** acarological research, *Amblyomma dubitatum*, *Amblyomma sculptum*, *Hydrochoerus hydrochaeris*, riparian forests, Rickettsia spp

## Abstract

**Aim:**

The vulnerability of tropical developing countries to the emerging disease constitutes a critical phenomenon in which the invasion of wild niches by human hosts, contributes to expansion of zoonotic diseases, such as the Brazilian spotted fever (BSF). This study performed a diagnosis of species occurrence of their hosts (*Hydrochoerus hydrochaeris*) and vectors (*Amblyomma sculptum* and *Amblyomma dubitatum*) on the warning area for this reemerging disease in Brazil.

**Materials and Methods:**

The study was conducted in a warning area for BSF in the city of Americana, São Paulo state. The occurrence of capybaras was registered by use of binoculars and GPS equipment and 24 acarological researches were performed through 180 CO2 traps. Samples of adult ticks were dissected for salivary glands removal, DNA extraction, and evaluation by polymerase chain reaction (PCR) being tested by initial *gltA*-PCR, *ompA*-PCR, and *Rickettsia bellii*-specific PCR, with the positive samples subjected to sequencing.

**Results:**

Eleven clusters of capybaras (total of 71 individuals), were observed along the riparian of Ribeirão Quilombo and 7,114 specimens of *A. sculptum* and 7,198 specimens of *A. dubitatum* were collected in this same area. About 568 samples of adult ticks were dissected for salivary glands removal, DNA extraction and evaluation by *gltA*-PCR, with results of 1.94% (11/568) of positive samples. Results for the initial *gltA*-PCR indicated none positive sample to *Rickettsia* species into *A. sculptum* and 11 positive samples to *A. dubitatum*. These samples were negative to the *ompA*-PCR and positive to the *Rickettsia bellii*-specific PCR protocol and subjected to DNA sequencing, whose result indicated 100% similarity to *Rickettsia bellii*. The distribution of tick species *A. sculptum* and *A. dubitatum* was configured regarding to the biotic potential of the riparian areas, measuring the risks for BSF in peri-urban areas of Americana.

**Conclusion:**

These results confirmed a status of epidemiological warning with a strong association of the amplifiers hosts of *Rickettsia* and tick vectors for the transmission of BSF to humans in this region.

## Introduction

The Brazilian spotted fever (BSF) is a disease from compulsory notification, with incidence and prevalence associated to risk of human parasitism by tick vectors, which makes acarological surveillance, be extremely important for efficient control of this disease [1]. Their etiologic agent (*Rickettsia rickettsii*) presents large pathogenicity and genetic variability, with more virulent circulating genotypes, establishing wide variation in lethality rates [2] and although determine low morbimortality in the population is among the most lethal etiologic agent of all known infectious diseases [3].

Their transmission occurs exclusively by ticks and in the São Paulo state, *Amblyomma sculptum* in the central part and *Amblyomma aureolatum* in the eastern part, has been implicated in the transmission of *R. rickettsii* to humans [4].

In the period 2003-2008, there was a significant expansion of the transmission areas, with the occurrence of 240 cases and 71 deaths in urban and peri-urban areas, with a lethality rate of 21.9-40% in the state of São Paulo, demonstrating deep modification in the eco-epidemiological characteristics of this disease [5].

Microbiological infections acquired from animals, known as zoonoses, have an enormous risk to public health, where 60% of emerging human pathogens are zoonotic, and more than 71% of these have their origin in wildlife. These pathogens can switch hosts for the acquisition of new genetic combinations that alter its pathogenic potential or by changes in behavior or socioeconomic, environmental, or ecological characteristics of hosts. Changes from natural habitats generate ecological imbalances that determine significant modifications in the diversity of interactions between reservoirs, hosts and vectors, increasing the chance of transmission of this disease [6], where the potential distribution of capybara is associated with anthropogenic environments, particularly by the intensive agricultural use [7].

At the same time, the effects of climate change in the ecology of landscapes resulted in an expansion of the number of ticks and biological hosts, with the consequent expansion of areas of risk to human or animal health in recent years [8].

The emergence and reemergence of diseases transmitted by ticks reached an overall increase of high significance as biological phenomena characterized by changes on environmental and human behavior [9] in consequence to the territorial mixture of wildlife, domestic animals and humans, combined with habitat fragmentation and increasing of circulation pathogenic microorganisms from natural areas to humans [10].

In the case of BSF, another aggravating factor in its epidemiology is the biological characteristic of *A. sculptum* as true reservoir of rickettsiae in nature, where all evolutionary stages are able to remain infected, ensuring a focus of prolonged disease transmission. In general, the tick *A. sculptum* is the most prevalent species in domestic and wild animals and in the environment, being the main vector specie to establish a bridge to pathogenic bioagents between domestic and wild fauna. The human parasitism by *A. sculptum* is common, because of their aggressiveness toward humans, being the principal vector of *Rickettsia rickettsii*, the etiologic agent of lethal spotted fever in Brazil [11].

The capybara (*Hydrochoerus hydrochaeris*) can be used as a sentinel for rickettsial diseases, as the BSF, seen evidencing the circulation of rickettsiae suggesting their involvement in the life cycle of this bacterium [12-14]. An overpopulation of capybaras in certain endemic areas of BSF will be accompanied by a high environmental infestation for all parasitic stages of *A. sculptum* and *A. dubitatum* [9,15]. Therefore, capybaras in urban areas should be considered synanthropic species and effective methods of population control need to be developed [16]. Is there any relationship between the increase in capybara populations and the reemergence of the disease in many areas at São Paulo State, since the capybaras population and the number of cases of BSF increased significantly during the past three decades [17].

Capybaras are social mammals (large rodents) widely distributed in riparian habitats and much favored by the landscape alteration in a watershed anthropogenically modified, with their strong relationship with water as a resource for many activities turned streams and rivers into corridors of intermittent flow, where competition for water and food, under a social and gregarious intrinsic territoriality may provide a strong relationship with the dispersion mechanisms [18]. Seasonal fluctuations in the amount of food available exert impacts on ecological aspects of the behavior of the capybara, conditioning the group size and the increased frequency of young individuals [19].

The impacts generated by the expansion of sugarcane in São Paulo on biodiversity of wildlife, on environment and on public health have shown an increase in the abundance of rodents and eutrophication of their aquatic ambience resulting in the appearance of infectious diseases associated with wildlife, as the hantavirus, leptospirosis, and spotted fever in humans. Sugarcane plantations have a lower diversity of wild species, but significantly greater abundance of rodents that other local ecosystems, because crops of sugar cane can provide a considerable amount of food for capybaras (*H. hydrochaeris*) who have experienced a large increase in its population, associated with the expansion of sugar cane in the center-east of the state of São Paulo and whose growth has been associated with the local resurgence of spotted fever [20].

Epidemiological observations indicate that the capybara (*H. hydrochaeris*) is not a potential reservoir for BSF, no presenting the clinical signs of disease, but acting as an excellent amplifier host, disseminating the bacteria *R. rickettsii* in nature and infecting about 30% of the ticks during period of the rickettsemia [12,21]. These correlations admit the possibility of that imbalances promoted by the intervention of environmental agencies in the protection and preservation of capybaras groups, generate rapid recovery of this species that, without natural predators and into natural habitat degraded, becomes a synanthropic population in urban areas, increasing risks to BSF cases in the public parks and municipal dams.

Capybaras are very abundant in endemic areas for BSF, acting as primary hosts for all parasitic stages of *A. sculptum* and *A. dubitatum*, and as a competent amplifier host for *R. rickettsii*. This study in Americana City, São Paulo State, performed a diagnosis of species occurrence of their hosts (*H. hydrochaeris*) and vectors (*A. sculptum* and *A. dubitatum*) on the warning area for this reemerging disease in Brazil.

## Materials and Methods

### Ethical approval

This study was subject to the regulations of Publics Policies and Ethics Committee in Brazil by authorization nº 20562 for activities with scientific purpose, issued by the System of Authorization and Information on Biodiversity (SISBIO) of Chico Mendes Institute for Biodiversity Conservation (ICMBio).

### Study area

**Figure-1:**
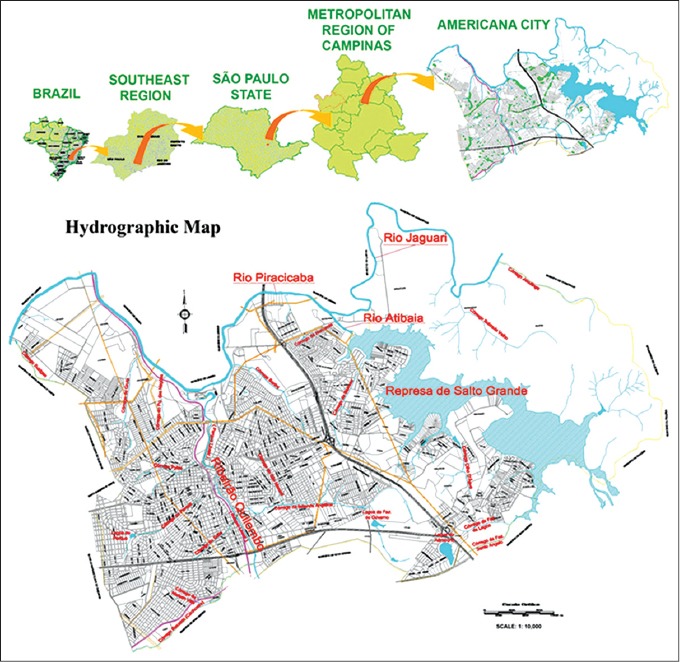
Map of Americana presenting their rich hydrography highly favorable for the density of capybaras into their territory.

According to epidemiological criteria, the study was conducted in a warning area for BSF in the city of Americana (22°44’21” S and 47°19’53” W), situated in a hydrographic basin composed for a dam and four rivers, in the state of São Paulo. This area located in an urban region of the municipality with a population 2,500 residents is characterized by riparian forests along 7,650 meters of the Ribeirão Quilombo. Americana is a municipality located in the eastern part of the state of São Paulo, southeastern Brazil, with a population of 224,551 inhabitants [22]. The existence of a rich hydrography created a highly favorable environment for the density of capybaras in their territory (Figure-1).

### Observation of capybara groups

The staff at surveillance and control ticks from the Department of Health from Americana, passing through the whole course of Ribeirão Quilombo conducted a situational diagnosis of capybara groups distributed along the riparian areas and began an observation of population groups by use of optical instruments (Romitar binoculars with zoom ranging from 15 to 180 times, 100 mm lens and view field of 52 m/1000 m) and GPS equipment (Garmin eTrex Summit HC).

The occurrence of capybaras in these locations was verified through detection of footprints and traces of faeces to localization and observation of individuals.

### Acarological research

**Figure-2:**
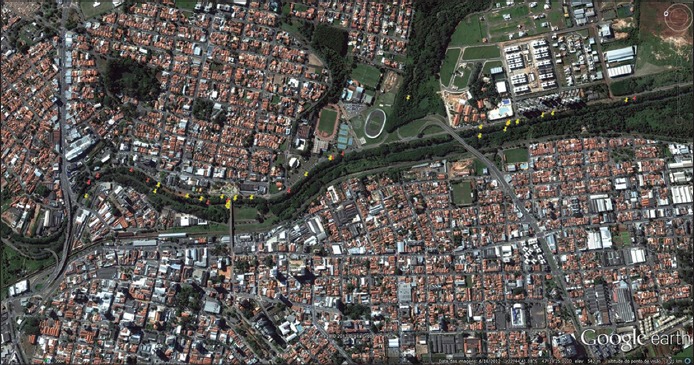
Spatial distribution of the clusters of capybaras (red mark) and acarological researches (yellow mark), georeferenced at different points in the riparian forest of Ribeirão Quilombo. Americana, São Paulo, Brazil.

About 24 acarological researches were distributed into 180 CO_2_ traps, at different points of riparian forest of Ribeirão Quilombo (Figure-2). The free-living ticks were collected after 1 h in the traps containing 800 g of dry ice in weekly repeated procedure between 09:00 am and 12:00 am [23] and were examined under a stereoscopic microscope for adults identification, sexed and classified taxonomically according to dichotomous keys translated and modified [24].

The collected larvae were identified by morphological criteria, and differentiated as the largest body size, oval outline in *A. dubitatum*, in comparison to *A. sculptum* [25] and nymphs characterized by taxonomic identification key of *Amblyomma* species [26].

### Molecular analysis

All adult specimens of ticks collected were placed in 1.5 mL microtubes and stored at -80°C, for DNA extraction and polymerase chain reaction (PCR) analysis. To evaluate the potential infectivity for *Rickettsia*, each adult ticks was thawed and subjected to identification, separation and dissection to removal of salivary glands under a stereomicroscope [27] for DNA extraction, through the GT protocol (chloroform and guanidine isothiocyanate) [28] and tested by *Rickettsia* genus-specific PCR using primers CS-62/CS-462, targeting a 401-bp fragment for citrate synthase gene of *Rickettsia* spp. (*gltA*) [29].

The PCR has been used for detection of *Rickettsia* spp. in gross crushed ticks, by amplification of *gltA* gene fragment, being positive samples for this gene subjected to amplification of *rOmpA* gene for identification of spotted fever group rickettsiae (SFG). The PCR followed by sequencing gene permits identification and phylogenetic analysis of different species of rickettsiae. Through PCR can be assessed several specific genes of rickettsiae such as 16S rRNA, the *gltA* (citrate synthase), found in all species of rickettsiae [29] and genes that encode proteins *OmpA* and *OmpB*, important in the pathogenesis of SFG rickettsiae.

The *gltA*-positive samples were tested by two others PCR, one assay using primers Rr190.70 p/Rr190.602n, which amplified a 532-pb fragment for the outer membrane protein gene of *Rickettsia* (*ompA*) which is specific for the SFG [30], and another assay specific to *Rickettsia bellii*, using the primers 5′-ATCCTGATTTGCTGAATTTTTT-3′ (forward) and 5′-TGCAATACCAGTACTGACG-3′ (reverse), which amplified a 338-bp fragment of the *R. bellii gltA* gene [31]. The amplified material was purified by ExoSAP-IT^®^ (USB^®^ Corporation) and subjected to sequencing using “BigDye Kit 3.1” and the DNA sequencer ABI Model “PRISM 3100 Genetic Analyzer” (Applied Biosystems, Foster City, CA, USA). The sequences obtained were submitted to the “BLAST analysis” software (National Center for Biotechnology Information, Bethesda, MD, USA) to determine the similarities of the partial rickettsial sequences generated in the current study [32].

## Results

14,312 ticks (7,114 specimens of *A. sculptum* and 7,198 specimens of *A. dubitatum*) were collected and a total of 681 adult specimens of *A. sculptum* (301 males and 380 females) and 658 adult specimens of *A. dubitatum* (320 males and 338 females) were identified. 2,447 nymphs and 3,986 larvae of *A. sculptum* and 1,160 nymphs and 5,380 larvae of *A. dubitatum* were identified.

**Figure-3:**
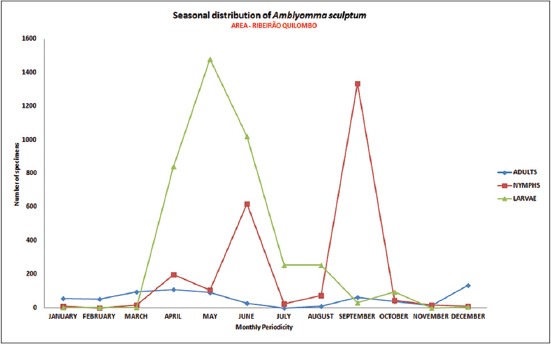
Seasonal distribution of the population of adults, nymphs and larvae of *Amblyomma sculptum*. Riparian forest. July 2009 to June 2010. Warning area for Brazilian spotted fever. Ribeirão Quilombo, Americana, São Paulo, Brazil [25].

**Figure-4:**
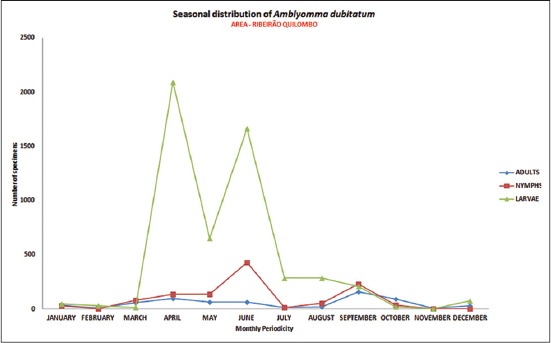
Seasonal distribution of the population of adults, nymphs and larvae of *Amblyomma dubitatum*. Riparian forest. July 2009 to June 2010. Warning area for Brazilian Spotted Fever. Ribeirão Quilombo, Americana, São Paulo, Brazil [25].

The distribution of seasonal patterns of *A. sculptum* (Figure-3) and *A. dubitatum* (Figure-4) were characterized by the presence of adults and immatures during all months of the period, in the areas of riparian forest of Ribeirão Quilombo analyzed at the municipality of Americana.

The human infestation was actively observed by adults, nymphs and larvae of *A. sculptum* and *A. dubitatum* in all acarological research conducted in the warning area studied, through the human parasitism record in workers staff involved in this activity.

At epidemiological point of view, depending on the relation between vectors, their hosts and the occurrence of human infestation in the area of riparian forest surveyed, it was observed a higher prevalence of *A. dubitatum* (58.4%) against *A. sculptum* (41.6%), associated to respective presence of capybaras, opossums, horses and dogs observed in this area.

568 samples of adult ticks were dissected for salivary glands removal, DNA extraction and evaluation by *gltA*-PCR, with results of 1.94% (11/568) of positive samples. Analyzing the results by species, no positive results for *A. sculptum* and 3.63% (11/303) of samples to *A. dubitatum* were positive.

The 11 samples positive for the initial *gltA*-PCR were negative to the *ompA-*PCR and positive to the *Rickettsia bellii-*specific PCR protocol. PCR products were subjected to DNA sequencing, where all the sequences obtained showed 100% similarity to the corresponding sequence of *R. bellii* in GenBank (accession number CP000087).

Eleven clusters of capybaras (represented by two different couples, two satellite females, and seven familiar social groups) were observed and identified at 7.65 km of riparian vegetation along Ribeirão Quilombo (Figure-2). Each social group ranged from 03 to 19 individuals. Three adult males, 20 adult females, 20 juvenile females, and 28 cubs in a total of 71 individuals were observed, and were mapped 57 shelters, refuges, or rest areas; 12 transit areas defined by tracks with footprints and 08 grazing areas.

## Discussion

The seasonal activity of free-living stages seeking a host, and the population density of ticks that perform human parasitism, can directly affect the occurrence of BSF in a region, for there are also seasonal prevalence for disease, as well as the stages of the tick, where most cases occur in the months from September to November (spring and early summer), more frequent at the time of predominance of larvae and nymphs compared to adults ticks that have lower predilection for parasitism in humans.

Regarding the main vector of BSF, recent studies reassessed the taxonomic status of *Amblyomma cajennense* through morphological and molecular analyzes, indicating a complex of six species (*A. cajennense* sensu stricto Fabricius, 1787, *Amblyomma tonelliae* Nava, Beati and Labruna, 2014, *Amblyomma interandinum* Beati, Nava and Cáceres, 2014, *Amblyomma patinoi* Labruna, Nava and Beatos, 2014, *Amblyomma mixtum* Koch, 1844 and *A. sculptum* Berlese, 1888 [33]. As a result, two species of ticks have been validated for Brazil (*A. cajennense* sensu stricto and *A. sculptum*) within the complex *A. cajennense*, causing the new name of *A. sculptum* for the species in the state of São Paulo. Therefore, the *A. sculptum* species presents the capybara (*H. hydrochaeris* Linnaeus, 1776) as a primary host for all your parasitic stages (adults, nymphs, and larvae) [14].

*A. sculptum* presents an annual pattern generation in the Southeast region of Brazil, with the three stages markedly distributed throughout the year. The larvae occur primarily between the months from March to July. The nymphs between the months from July to November, and adults predominantly between the months from November to March controlled by behavioral diapause during one phase of the life cycle [34], also having great interference of photoperiod and climatic variables on the level of environmental infestations studied.

Based on analysis of the scientific literature, the human parasitism by *A. sculptum* was reported in 23 municipalities and by *A. dubitatum* in three municipalities of the state of São Paulo [35]; and during the last 10 years in the state of São Paulo, the number of confirmed cases of BSF by location was similar among endemic areas, where *A. aureolatum* or *A. sculptum* were the vectors [36].

Considering the seasonal distribution analysis, the prevalence of species of *A. sculptum* and *A. dubitatum*, and the epidemiological profile established with the incidence of ten cases of BSF and six deaths in humans in the respective study areas, from 2004 to 2013, was proven the epidemiological risk of active transmission of this zoonosis in the municipality of Americana, São Paulo state [25]. All these cases were related to tick bites acquired along the water courses of the municipality, where there are established populations of capybaras, with an association very alarmingly, considering that human infestation by ticks has become very common in these urban areas, with a significant population susceptible to the highlighted risks.

The increase in capybara populations during the last decades may be related to the reemergence of BSF in many areas in São Paulo State. Besides the transmission risks of this zoonosis to communities and workers in these areas, we know that its main natural host, the capybara (*H. hydrochaeris*) are very selective about the food, competing with cattle in pastures, creating a common and narrow convivial with these farm animals and acting as an important wild and rural reservoir of diseases and an excellent amplifier of arthropod vectors, with significant performance and important mechanisms and flows for sustainability related to disease cycles in Animal and Public Health, and its interrelationships in economic losses in livestock breeding and commercial exploitation of domestic livestock species [37].

The increasing expansion of cities have pressured the wildlife animals to live adapted in fragments of green areas threatened by the continuous reduction on natural areas [38].

The increase in capybaras populations in small riparian forest or in lakes and dams forest fragments (characterizing environmental protection areas in agricultural production units) is associated with unbearable levels of environmental infestations by ticks from the species *A. sculptum* and *A. dubitatum*. Humans and domestic animals in such places are constantly exposed to parasitism and the risk of tick-borne diseases, such as the BSF [39].

The behavioral changes observed in these individuals (gregarious animals with agonistic interactions in respect to dominance hierarchy), due to the loss of their natural sense of preservation (flight distance from) to humans caused an increase in reporting of human parasitism by ticks, due to common frequency of these animals hosts, in these urban areas.

The infection of capybaras (*H. hydrochaeris*) by *R. rickettsii* and its role as amplifier host for the horizontal transmission of *R. rickettsii* to *A. sculptum* tick was evaluated by extraction of DNA samples of ticks, followed by real-time PCR targeting the *gltA* gene from *Rickettsia* and detection of antibodies to *R. rickettsii* by IFA in blood serum samples of capybaras and guinea pig [12]. As a result, 20-35% of the ticks fed in tested groups of capybaras became infected, indicating that *R. rickettsii* was capable of infecting capybaras without clinical harm to their host, but inducing a bacteremia able to cause infection in guinea pig and ticks, indicating that *H. hydrochaeris* truly acts as an amplifier host of *R. rickettsii* to *A. sculptum* in Brazil.

A case study was presented where the focus of concern of management in a protected area shifted from a clear public condemnation of a wild species (capybara), for a proposed landscape management and communication in order to reduce the exposure of people to ticks, reducing risks of diseases associated with them. Forms of *Rickettsia* found did not belong to the SFG, but this information did not reduce the concern for health monitoring and care with exposure to ticks [40].

Unexpected changes in distribution and abundance of species has often been attributed to the complexity of nature, where many of the ecological surprises we have faced over the centuries (pandemics, population collapse of species, and major changes in ecosystems) were caused or favored by the extinction of predating species and/or the introduction of wild species for economic exploitation [41].

Despite an epidemiological characterization of warning and a prevalence of 1.94% to *R. bellii* in the studied area, the results derived from molecular analyzes, no showed rickettsiae of the SFG, in species of *A. sculptum* and *A. dubitatum*, probably due to deleterious effect caused by *R. rickettsii* in these ticks [42].

## Conclusion

Thus, the work of monitoring of this population of circulating capybaras in Ribeirão Quilombo, serological surveys performed in sentinel animals and bioecological studies of ticks with information about their population dynamics, related to the host and the environment, can ensure the implementation of an effective program to arthropods control and prevention of BSF, through monitoring by the molecular diagnostic tools in perfect sync with the instruments of epidemiological and serological surveillance, intensified by an active acarological surveillance and a syndromic surveillance for human patients suspected.

## Authors’ Contributions

The observation of capybara groups, acarological researches, taxonomic identification of ticks and molecular analysis were conducted by JBN and JB. Data were collected and interpreted by JBN. The manuscript was prepared jointly by JBN and KMRD. All authors read and approved the final manuscript.
